# The burden and predictors of venous thromboembolic diseases in patients with multiple primary malignancies

**DOI:** 10.1002/cnr2.1742

**Published:** 2022-10-31

**Authors:** Moustafa S. Alhamadh, Rakan B. Alanazi, Muhannad Q. Alqirnas, Abdulrahman Yousef Alhabeeb, Yusra Sajid Chachar, Mohammad Alkaiyat, Fouad Sabatin

**Affiliations:** ^1^ College of Medicine, King Saud bin Abdulaziz University for Health Sciences (KSAU‐HS) Ministry of the National Guard‐Health Affairs Riyadh Kingdom of Saudi Arabia; ^2^ King Abdullah International Medical Research Center Ministry of the National Guard‐Health Affairs Riyadh Kingdom of Saudi Arabia; ^3^ College of Sciences and Health Professions at King Saud Bin Abdulaziz University for Health Sciences (KSAU‐HS) Ministry of the National Guard‐Health Affairs Riyadh Kingdom of Saudi Arabia; ^4^ Department of Medical Oncology King Abdulaziz Medical City, Ministry of the National Guard‐Health Affairs Riyadh Kingdom of Saudi Arabia

**Keywords:** cancer‐associated thrombosis, multiple primaries, multiple primary malignancies, thromboprophylaxis, venous thromboembolism

## Abstract

**Background:**

Venous thromboembolism (VTE) represents a considerable burden on cancer patients' survival and quality of life, but this burden varies based on the patient's baseline characteristics and cancer‐related factors. Although solid evidence on the predictors and effect of VTE in cancer patients exists.

**Aim:**

To evaluate VTE rate, morbidity, and mortality to develop parameters that could predict VTEs and their associated mortality in patients with multiple primary malignancies (MPMs).

**Method and Results:**

This was a retrospective cohort study that took place at King Abdulaziz Medical City, Riyadh, Kingdom of Saudi Arabia. Two hundred and forty‐two patients with at least two biopsy‐proven malignancies and had at least 3 months of follow‐up after MPMs diagnosis were included. VTE was diagnosed in 14.5% of the cases, two‐thirds of which were deep vein thrombosis. VTE was significantly associated with a higher mortality and worse survival. Predictors of VTE after MPMs diagnosis were a high ECOG performance status at MPMs diagnosis, a metastatic first primary malignancy, and ICU admission after MPMs diagnosis. Having a GI or hematological malignancy as the second primary malignancy, a high D‐dimer at ICU admission, and palliative care referral were significantly associated with a higher mortality in patients who had VTE.

**Conclusion:**

VTE was diagnosed in 14.5% of patients with MPMs and it significantly compromises their survival. We believe that these results might be of particular benefit since the phenomenon of MPMs is becoming more frequently encountered.

## 1 INTRODUCTION

Venous thromboembolism (VTE), including deep vein thrombosis (DVT) and pulmonary embolism (PE), represents a major public health burden with an estimated annual incidence of 0.5% compared with 0.1% in the general population, and it carries with it increased morbidity and mortality.^1^, ^2^, ^3^ Approximately, 300 000 deaths are attributed to VTE annually, 20% of which occur in cancer patients.^4^, ^5^ VTE poses a remarkable financial burden on the health care system and significantly impairs the patients' quality of life, as up to half of DVT patients develop post‐thrombotic syndrome causing pain, swelling, and ulcerative skin changes, and decreases patients' survival, as PE has a 30‐day mortality that reaches up to 30%.^6^, ^7^

Thrombosis susceptibility is largely attributed to endothelial damage, flow stasis, and hypercoagulation, collectively known as the Virchow's triad.^8^, ^9^ In addition to the genetic variants that predispose VTE such as factor V Leiden, complete plasminogen activator inhibitor 1, antithrombin, and protein C and S deficiencies, prothrombin gene mutation, and fibrinogen gamma,^10^, ^11^, ^12^ provoking and non‐provoking environmental factors, including cancer, trauma, immobility, surgery, pregnancy, age, gender, ethnicity, obesity, oral contraceptive, and corticosteroid are also implicated.^13^, ^14^

The risk of VTE in cancer patients can be sixfold higher than in patients without cancer, especially in the first year of diagnosis, and metastasis increases the risk by 20‐fold.^15^, ^16^ With an annual incidence rate ranging from 3% to 15%, VTE notably complicates cancer's course and increases its mortality by more than threefolds.^5^ This incidence rate is highest with gastric cancer, pancreatic cancer, and multiple myeloma.^17^ Regardless of cancer type, cancer is considered a prothrombotic state because of tumor‐related factors such as the tumor's primary site, stage, and grade, and the patients' needs for multiple surgical interventions, frequent hospitalization, immobilization, infection, central and peripheral vascular catheters, chemotherapy such as Cisplatin, Fluorouracil, and Cetuximab, anti‐angiogenesis agents such as Bevacizumab and Sunitinib, hormonal therapy such as Tamoxifen and aromatase inhibitors, and radiotherapy.^18^, ^19^, ^20^, ^21^

Thromboprophylaxis is not generally recommended for all cancer patients as they are more subjected to bleeding.^22^ Therefore, a meticulous decision for thromboprophylaxis should be taken at both the high‐risk patients and at the most appropriate times.^5^ Knowledge about cancer‐associated thrombosis allowed the development of multiple risk stratification models, such as the Khorana, Vienna, and COMPASS‐CAT scores, to predict VTE and identify cancer patients who might need frequent or regular VTE screening and benefit from thromboprophylaxis.^23^, ^24^, ^25^ For example, the Khorana score utilizes simple clinical and laboratory parameters to stratify cancer patients into low‐, intermediate‐, and high‐risk patients.^23^

Owing to the advancement in screening, prevention, diagnosis, and therapeutics, cancer‐related mortality has declined by 29% from 1991 to 2017, increasing the patients' overall survival and life expectancy, which exposes them to more mutations and epigenetic changes.^26^ At the expense of that, the phenomenon of multiple primary malignancies (MPMs) has been frequently encountered with an incidence ranging from 0.52% to 11.7%, and is defined as the occurrence of two or more independent malignancies in the same or different organs.^27^, ^28^ Because of these facts and since no studies concerning VTE in MPMs are available, we aimed to evaluate the rate, morbidity, and mortality of VTEs and their effect on the survival of patients with MPMs, and to develop predictors for the development of VTEs and their associated mortality.

## 2 METHOD

### 2.1 Objectives

To evaluate the rate, morbidity, and mortality of VTEs and their effect on the survival of patients with MPMs, and to develop predictors for the development of VTEs and their mortality.

### 2.2 Study design and setting

This was a single‐center retrospective cohort study conducted at King Abdulaziz Medical City (KAMC), Department of Medical Oncology, Ministry of the National Guard‐Health Affairs, Riyadh, Kingdom of Saudi Arabia. KAMC is an academic government‐funded tertiary hospital that combines clinical care, training, academics with research, and state‐of‐the‐art medical technologies.

### 2.3 Inclusion and exclusion criteria

All the patients with at least two biopsy‐proven malignancies from 2016 to 2022 were included. c. Patients with missing or uncertain data, lost to follow‐up for a long time, referred to our institution for only diagnostic workup, benign tumors, or suspected malignancies, lack of pathology report or indecisive pathology report, were excluded.

### 2.4 Sample size and data collection

Initially, the Department of Medical Oncology tumor registry identified a total of 296 patients with MPMs from 2016 to 2022. After applying the aforementioned inclusion and exclusion criteria, 242 patients were included. The required data were obtained by screening all the oncology electronic records (via the KAMC electronic system “Best‐Care”). The following data were collected: demographics, comorbidities, chronic steroid use, first, second, and third malignancy anatomic location, histopathologic morphology, and treatment (chemotherapy, radiotherapy, surgery), laboratory values such as hemoglobin, platelets, CRP, ESR, and D‐dimer at MPMs diagnosis, Eastern Cooperative Oncology Group (ECOG) performance status at MPM diagnosis and last visit, intensive care admission, referral to palliative care, survival status, and VTE‐related data (date of diagnosis, location, and treatment). Synchronous malignancy was defined as a second malignancy that occurs either simultaneously, or within 6 months after the first malignancy diagnosis, and a metachronous malignancy as a second malignancy at least 6 months after the first malignancy diagnosis. DVT and PE were considered if a thrombus was seen in Doppler ultrasound or chest CT angiography, respectively.

### 2.5 Statistical analysis

The Statistical Package for the Social Sciences (SPSS version 22) was used for data analysis. The categorical variables are presented as frequency and percentage, and the numerical variables as mean ± standard deviation. The time intervals between the first and second primary diagnoses and between the second primary and VTE diagnoses were presented as median and percentiles. Chi‐square or Fisher's Exact test was used to test the association between categorical variables, and independent sample *t* test or Mann–Whitney test was used to test the association between numerical variables of patients who had VTE and who had not. To develop predictors for VTE, univariate and multivariate logistic regression models were done to estimate odds rations and 95% confidence intervals for the binary outcomes. Only variables with a *p* < .2 between the two groups were included in the multivariate model. The survival probabilities of patients with VTE were compared with patients without VTE using the Kaplan–Meier method. The log‐rank test was used to compare the survival time between different groups (VTE vs. Non‐VTE and long‐term prophylactic anticoagulants vs. no long‐term prophylactic anticoagulants). A test was considered significant if two‐sided *p* < .05.

## 3 RESULTS

### 3.1 Baseline characteristics, demographics, and comorbidities

**FIGURE 1:**
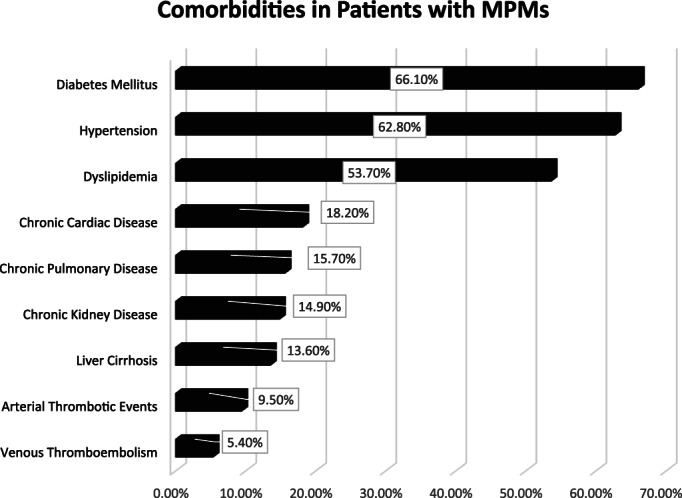
Comorbid conditions in patients with multiple primary malignancies (MPMs) in consecutive order. Diabetes mellitus and hypertension were the most common comorbidities, followed by dyslipidemia, chronic cardiac disease, and chronic pulmonary disease. Only a history of venous thromboembolism prior to MPMs diagnosis, which was observed in only 5.4%, was associated with venous thromboembolism (VTE)

**FIGURE 2:**
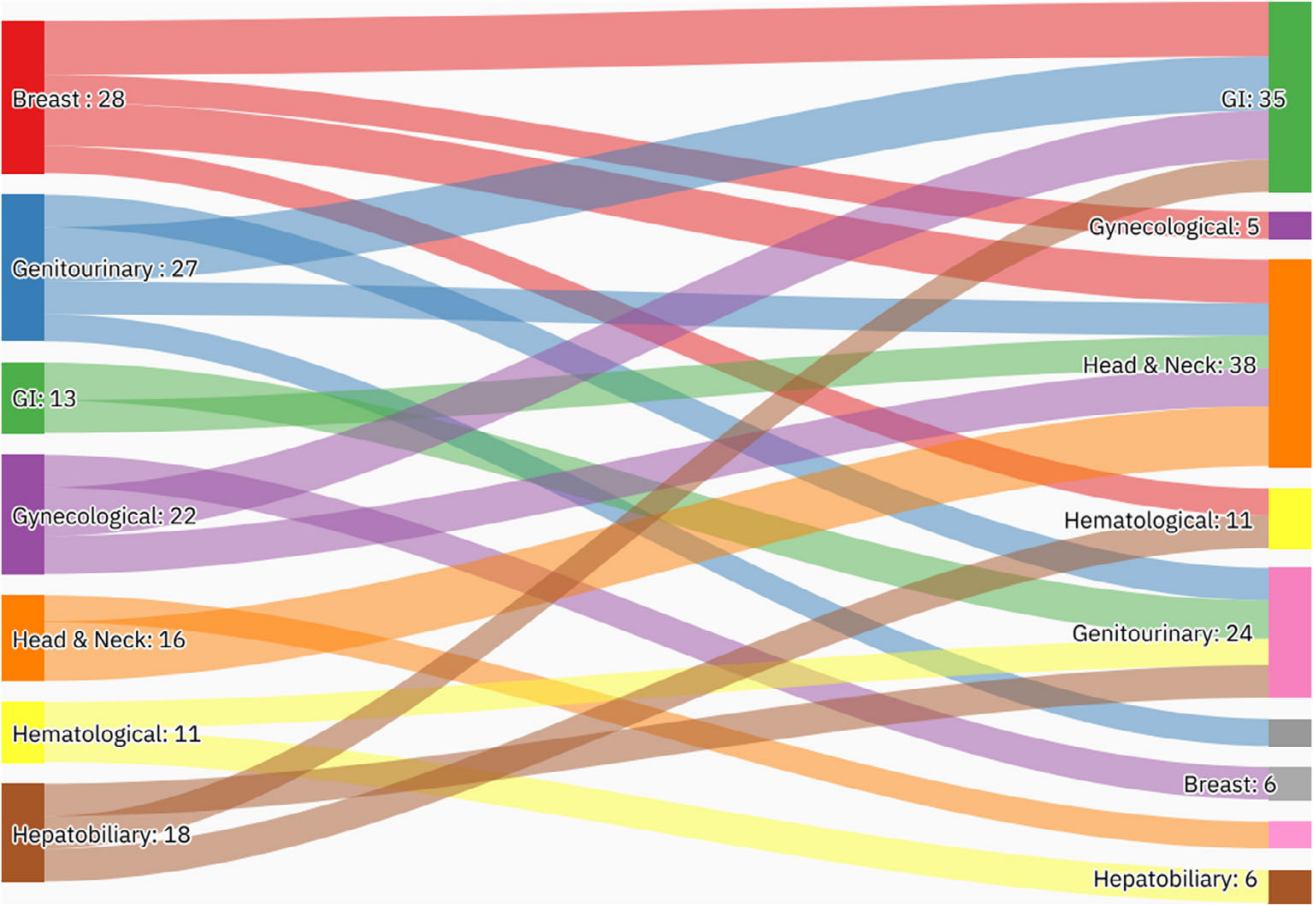
An alluvial plot showing the most frequent malignancy combinations. The most frequent malignancy combinations were head and neck—head and neck, breast—GI, genitourinary—GI, gynecological—GI, and breast—head and neck. The aim of this figure is to trace the pattern of the first and second malignancy combinations.

**FIGURE 3:**
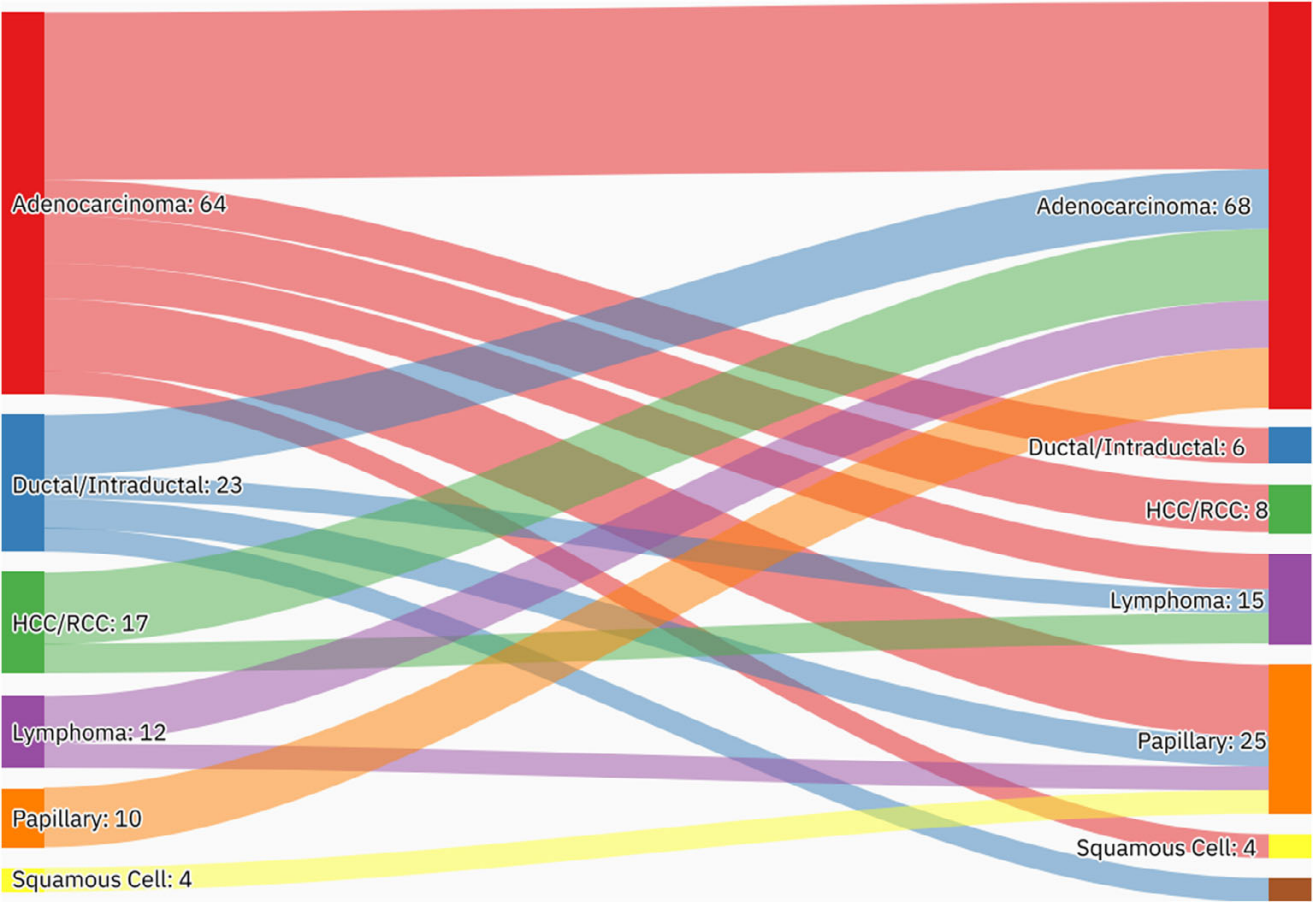
An alluvial plot showing the most frequent histopathological combinations. The most frequent histopathological combinations were adenocarcinoma—adenocarcinoma, adenocarcinoma—papillary carcinoma, and HCC/RCC—adenocarcinoma. The aim of this figure is to trace the pattern of histopathological combination of the first and second diagnoses.

A summary of patients' baseline characteristics and comorbid conditions is shown in (Table 1). There was a total of 296 patients with MPMs from 2016 to 2022, only 242 of whom were eligible. More than half (53.3%, *n* = 129) of the patients were female. The patients' mean age was 67.2 ± 12.5 years with an average BMI of 28.2 ± 7.4 kg/m^2^. The most notable associated comorbidities were diabetes mellitus, hypertension, dyslipidemia, chronic cardiac disease, chronic pulmonary disease, and chronic kidney disease, accounting for 66.1%, 62.8%, 53.7%, 18.2%, 15.7%, and 14.9%, respectively (Figure 1). Only a few patients had a family history of cancer (5.0%, *n* = 12) or a previous history of organ transplantation (3.3%, *n* = 8). Almost a quarter of the cases were smokers (21.5%, *n* = 52). The mean age at diagnosis of the first, second, and third primary malignancies was 59.4 ± 13.7, 63 ± 13, and 69.1 ± 8.7 years, respectively. More than a third of the cases were referred to palliative care (32.6%, *n* = 79). The overall mortality rate was 41.7%, with cardiopulmonary arrest (17.8%), and infection (7.0%) being responsible for most of the known causes of mortality. Almost three‐quarters (72.3%, *n* = 175) of the cases were metachronous and 27.7% (*n* = 67) synchronous, with an overall average diagnostic interval of 47.6 ± 58.2 and a median of 24 (Q1, Q3: 4.1, 71.9) months. The most frequent anatomical site of the first primary malignancy was GI cancer (14.9%), followed by breast (14.5%), genitourinary (13.6%), head and neck (12.0%), and hepatobiliary cancer (12.0%). Regarding the second primary malignancy, GI malignancies (22.3%) were the most frequent, followed by head & neck (20.2%), genitourinary (12.8%), hematological (10.3%), and hepatobiliary (9.1%) malignancies. Only 7.9% (*n* = 19) of the cases had triple primary malignancies, with GI (*n* = 7), thoracic (*n* = 5), and hepatobiliary malignancies being the most frequent third primary diagnoses. The most frequent histopathologic morphology of the first primary malignancy was adenocarcinoma (31.0%), followed by infiltrating ductal carcinoma (12.8%), renal cell carcinoma/hepatocellular carcinoma (11.2%), and papillary carcinoma (9.9%). The most frequent histopathologic morphology of the second primary malignancy was adenocarcinoma (36.0%), followed by papillary carcinoma (15.3%), renal cell carcinoma/hepatocellular carcinoma (8.3%), lymphoma, and squamous cell carcinoma (7.9% for both) (Table 2). The most frequent malignancy combinations (First primary malignancy—Second primary malignancy) were head and neck—head and neck (*n* = 11), breast—GI (*n* = 10), genitourinary—GI (*n* = 10), gynecological—GI (*n* = 9), and breast—head and neck (*n* = 8) (Figures 2 and 3).

**TABLE 1:** Patients' baseline characteristics and associated comorbid conditions

	*N*	%
Gender		
Male	113	46.7
Female	129	53.3
Age categories		
0–60 years	65	26.9
61–70 years	78	32.2
71–80 years	65	26.9
≥81 years	34	14.0
BMI categories		
Underweight	17	7.0
Normal	70	28.9
Overweight	64	26.4
Obese	91	37.6
Smoking status		
Smoker	52	21.5
Not smoker	190	78.5
Family history of malignancy		
Yes	12	5.0
No	230	95.0
History of venous thromboembolic disease prior to MPMs diagnosis		
Yes	13	5.4
No	229	94.6
Chronic use of antithrombotic medications		
Yes	38	15.7
No	204	84.3
Chronic steroid use		
Yes	26	10.7
No	216	89.3
Organ recipient		
No	234	96.7
Yes	8	3.3
Liver	6	2.5
Kidney	1	0.4
Bone Marrow	1	0.4
Number of primary malignancies		
2	223	92.1
>2	19	7.9
Type of MPMs based on the time interval		
Synchronous	67	27.7
Metachronous	175	72.3
ECOG performance status at MPMs diagnosis		
0	38	23.6
1	72	44.7
2	35	21.7
3	12	7.5
4	4	2.5
ECOG performance status at the last visit/prior to death		
0	25	14.6
1	68	39.8
2	27	15.8
3	30	17.5
4	21	12.3
Distant metastasis of first primary malignancy		
Yes	34	14.0
No	208	86.0
Distant metastasis of second primary malignancy		
Yes	29	12.0
No	213	88.0
Distant metastasis of third primary malignancy		
Yes	3	1.2
No	4	1.7
Chemotherapy		
Yes	183	75.6
No	59	24.4
Radiotherapy		
Yes	122	50.4
No	120	49.6
Surgical therapy		
Yes	218	90.1
No	24	9.9
Referral to palliative care		
Yes	79	32.6
No	163	67.4
Diabetes mellitus		
Yes	160	66.1
No	82	33.9
Hypertension		
Yes	152	62.8
No	90	37.2
Dyslipidemia		
Yes	130	53.7
No	112	46.3
Chronic cardiac disease		
Yes	44	18.2
No	198	81.8
Chronic pulmonary disease		
Yes	38	15.7
No	204	84.3
Chronic kidney disease		
Yes	36	14.9
No	206	85.1
Liver cirrhosis		
Yes	33	13.6
No	209	86.4
Arterial thrombosis^a^		
Yes	23	9.5
No	219	90.5
Intensive care unit admission		
Yes	46	19.0
No	196	81.0
Mechanical ventilation		
Yes	27	11.2
No	19	7.9
Vasopressors/inotropes		
Yes	34	14.0
No	12	5.0
Survival status		
Dead	101	41.7
Alive	141	58.3
Cause of death		
Infection/sepsis	17	7.0
Cardiopulmonary arrest	43	17.8
Multiorgan failure	13	5.4
Disseminated intravascular coagulation/massive bleeding	2	0.8
Cancer‐related non‐specific cause	25	10.3

Abbreviations: ECOG, eastern cooperative oncology group; MPMs, multiple primary malignancies.

^a^ Arterial thrombotic events include stroke and peripheral vascular disease but not coronary artery disease. Coronary artery disease, along with congestive heart failure, was included with “chronic cardiac disease.”

**TABLE 2:** Anatomical distribution of multiple primary malignancies and their histopathologic morphologies

	First malignancy *N* (%)	Second malignancy *N* (%)	Third malignancy *N* (%)	Total *N* (%)
Metastasis	34 (14.0)	29 (12.0)	3 (1.2)	66 (27.2)
Anatomical location	**First malignancy *N* (%)**	**Second malignancy *N* (%)**	**Third malignancy *N* (%)**	**Total *N***
Gastrointestinal	36 (14.9)	54 (22.3)	7 (2.9)	97
Breast	35 (14.5)	20 (8.3)	0	55
Head & neck	29 (12)	49 (20.2)	1 (0.4)	78
Thoracic	13 (5.4)	16 (6.6)	5 (2.1)	34
Genitourinary	33 (13.6)	31 (12.8)	1 (0.4)	65
Hepatobiliary	29 (12)	22 (9.1)	3 (1.2)	54
Hematological	27 (11.2)	25 (10.3)	2 (0.8)	54
Skin	11 (4.5)	10 (4.1)	0	21
Musculoskeletal	2 (0.8)	3 (1.2)	0	5
Gynecological	27 (11.2)	11 (4.5)	0	38
Histopathology	**First malignancy *N* (%)**	**Second malignancy *N* (%)**	**Third malignancy *N* (%)**	**Total *N***
Adenocarcinoma	75 (31.0)	87 (36.0)	10 (4.1)	172
Squamous cell	16 (6.6)	19 (7.9)	3 (1.2)	38
Adenosquamous	1 (0.4)	1 (0.4)	0	2
Papillary	24 (9.9)	37 (15.3)	1 (0.4)	62
Neuroendocrine	4 (1.7)	3 (1.2)	1 (0.4)	8
Ductal/intraductal	31 (12.8)	18 (7.4)	0	49
Follicular	1 (0.4)	3 (1.2)	0	4
Basal cell carcinoma	5 (2.1)	2 (0.8)	0	7
Leukemia	8 (3.3)	5 (2.1)	1 (0.4)	14
Lymphoma	19 (7.9)	19 (7.9)	1 (0.4)	39
Sarcoma	5 (2.1)	14 (5.8)	1 (0.4)	20
Teratoma/germ cell	6 (2.5)	2 (0.8)	0	8
RCC/HCC	27 (11.2)	20 (8.3)	1 (0.4)	48
Other	20 (8.3)	12 (5.0)	0	32

*Note*: Gastrointestinal includes stomach, small intestine, and colorectal cancers. Head and neck includes thyroid, nasopharynx, oral, and intracranial cancers. Genitourinary includes kidney, bladder, prostate, and testicular cancers. Hepatobiliary includes liver, bile system, and pancreas cancers.

Abbreviations: HCC, hepatocellular carcinoma; RCC, renal cell carcinoma.

### 3.2 Venous thromboembolism and associated mortality

**FIGURE 4:**
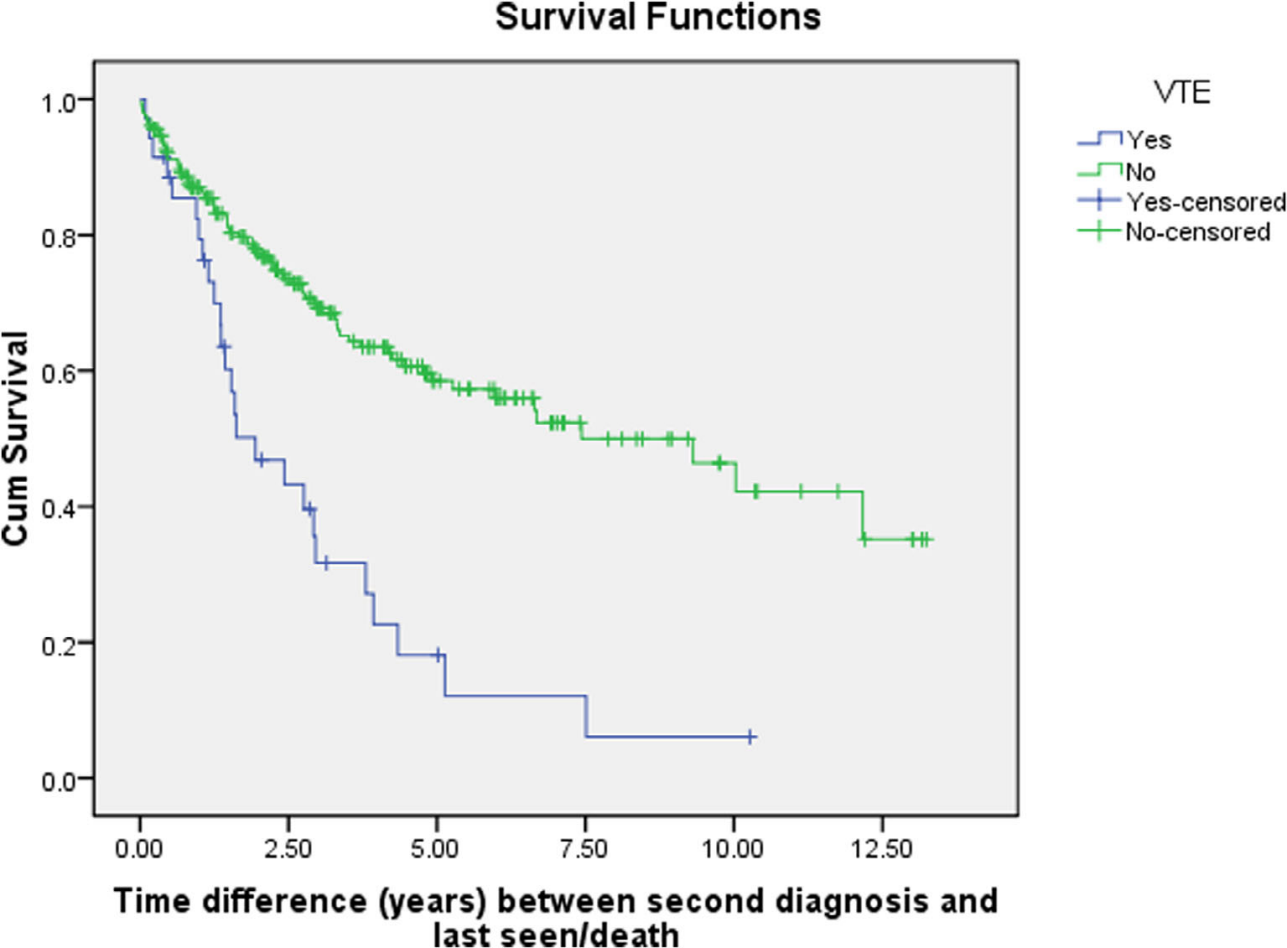
Survival of multiple primary malignancies (MPMs) patients with venous thromboembolism (VTE) and without VTE. The median survival time was 1.9 ± 0.6 and 7.4 ± 1.5 years for patients with VTE and without, respectively, and the difference was highly statistically significant (*p* < .001).

**FIGURE 5:**
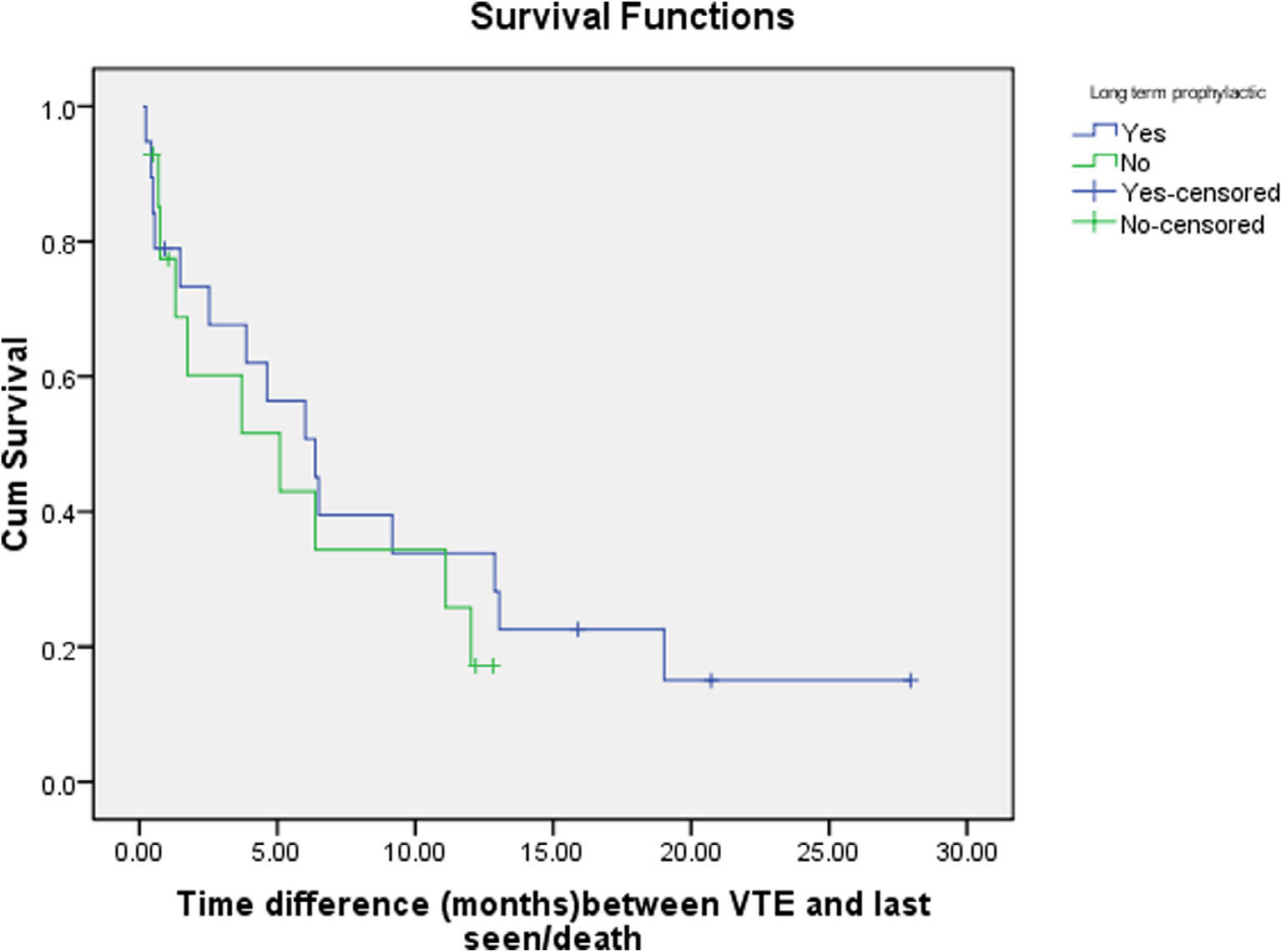
Survival of multiple primary malignancies (MPMs) patients who received long‐term prophylactic anticoagulant vs. patients who received long‐term prophylactic anticoagulant after therapeutic anticoagulant for venous thromboembolism (VTE). The median survival time was 6.4 ± 1.3 and 5.1 ± 2.8 months for patients who received and did not receive long‐term prophylactic anticoagulants, but the difference was not statistically significant (*p* = .472).

A summary of the locations and treatment of venous thromboembolic events is provided in (Table 3). VTE was diagnosed in 14.5% of the patients with an average time interval of 21 ± 25.6 and a median of 11.3 (Q1, Q3: 1.10, 32.07) months after the second primary malignancy diagnosis. Two‐thirds (60.0%, *n* = 21) of VTE cases were DVTs, two cases of which were bilateral. The left lower limb (*n* = 8) and right upper limb (*n* = 7) were the most frequent locations for DVT. There were 15 PE cases, 5 of which were bilateral. The most frequent locations for PE were lobar (*n* = 5) and segmental (*n* = 4) arteries. Only one patient had both DVT and PE, and a small proportion (5.4%, *n* = 13) of cases had a history of VTE prior to MPMs diagnosis. Patients with VTE had a significantly higher mortality rate (*p* < .001). Almost all (94.3%, *n* = 33) VTE patients received therapeutic anticoagulants, with enoxaparin (*n* = 21) being the most commonly used anticoagulant, followed by unfractionated heparin (*n* = 10) and DOAC (*n* = 2). Retrievable inferior vena cava filters were inserted only in three patients. More than half (57.8%, *n* = 19) were commenced on long‐term prophylactic anticoagulants, mainly enoxaparin (*n* = 12) and DOAC (*n* = 3). VTE after MPMs diagnosis was significantly associated with female gender (*p* = .027), being non‐smoker (*p* = .046), history of VTE prior to MPMs diagnosis (*p* = .005), high ECOG score at MPMs diagnosis (*p* = .003), meta‐static first primary malignancy (*p* = .012), low hemoglobin or high CRP level at MPMs diagnosis (*p* = .003, .036), chemotherapy (*p* = .001), and referral to palliative care (*p* < .001) (Tables 4 and 5). Having a GI or hematological malignancy as the second primary cancer (*p* = .039), high D‐dimer at ICU admission (*p* = .014), and referral to palliative care (*p* = .001) were significantly associated with higher mortality in patients who had VTE. The survival times were calculated from the date of VTE diagnosis to the date of death or date of the last follow‐up. The median survival time was 1.9 ± 0.6 and 7.4 ± 1.5 years for patients who had VTE and did not, respectively, and the difference was statistically significant (*p* < .001) (Figure 4). The median survival time was 6.4 ± 1.3 and 5.1 ± 2.8 months for patients who received and did not receive long‐term prophylactic anticoagulants, but the difference was not statistically significant (*p* = .472) (Figure 5).

**TABLE 3:** Locations and treatment of venous thromboembolic diseases after multiple primary malignancies diagnosis

	*N*	%
Deep vein thrombosis		
Yes	21	8.7
No	221	91.3
Bilateral	2	0.8
Location of deep vein thrombosis		
Right upper limb	7	2.9
Right lower limb	3	1.2
Left upper limb	3	1.2
Left lower limb	8	3.3
Pulmonary embolism		
Yes	15	6.2
No	227	93.8
Bilateral	5	2.1
Location of pulmonary embolism		
Main pulmonary	3	1.2
Lobar	5	2.1
Segmental	4	1.7
Subsegmental	3	1.2
Therapeutic anti‐coagulants		
Yes	33	13.6
No	2	0.8
Type of therapeutic anti‐coagulants		
Enoxaparin	21	8.7
Unfractionated heparin	10	4.1
Direct oral anti‐coagulant	2	0.8
Inferior vena cava filter insertion		
Yes	3	1.2
No	30	12.4
Long‐term prophylactic medication		
Yes	19	7.9
No	14	5.8
Type of long‐term prophylactic medication		
Enoxaparin	12	5.0
Unfractionated heparin	2	0.8
Direct oral anti‐coagulant	3	1.2
Warfarin	1	0.4
Aspirin	1	0.4

**TABLE 4:** Comparison between baseline characteristics and associated comorbid conditions in patients who had VTE versus patients who had not

	VTE		Non‐VTE		*p*‐value
	*N*	%	*N*	%	
Gender					
Male	10	8.8	103	91.2	.027
Female	25	19.4	104	80.6	
Age categories					
0–60 years	11	16.9	54	83.1	.804
61–70 years	12	15.4	66	84.6	
71–80 years	7	10.8	58	89.2	
≥81 years	5	14.7	29	85.3	
BMI categories					
Underweight	4	23.5	13	76.5	.365
Normal	9	12.9	61	87.1	
Overweight	6	9.4	58	90.6	
Obese	16	17.6	75	82.4	
Smoking status					
Smoker	3	5.8	49	94.2	.046
Not smoker	32	16.8	158	83.2	
Family history of malignancy					
Yes	2	16.7	10	83.3	1.000
No	33	14.3	197	85.7	
History of venous thromboembolic disease prior to MPMs diagnosis					
Yes	6	46.2	7	58.3	.005
No	29	12.7	200	87.3	
Chronic use of antithrombotic medications					
Yes	9	23.7	29	76.3	.084
No	26	12.7	178	87.3	
Chronic steroid use					
Yes	4	15.4	22	84.6	1.000
No	31	14.4	185	85.6	
Organ recipient					
No	1	12.5	7	87.5	1.000
Yes	34	14.5	200	85.5	
Type of MPMs based on the time interval					
Synchronous	7	10.4	60	89.6	.313
Metachronous	28	16.0	147	84.0	
ECOG performance status at MPMs diagnosis					
0	0	0.0	38	26.6	.003^a^
1	7	38.9	65	45.5	
2	10	55.6	25	17.5	
3	1	5.6	11	7.7	
4	0	0.0	4	2.8	
ECOG performance status at the last visit/prior to death					
0	0	0.0	25	16.9	.013^a^
1	7	30.4	61	41.2	
2	3	13.0	24	16.2	
3	9	39.1	21	14.2	
4	4	17.4	17	11.5	
Distant metastasis of first primary malignancy					
Yes	10	29.4	24	70.6	.012
No	25	12.0	183	88.0	
Distant metastasis of second primary malignancy					
Yes	5	17.2	24	82.8	.778
No	30	14.1	183	85.9	
Distant metastasis of third primary malignancy					
Yes	0	0.00	3	100.0	1.000^a^
No	1	25.0	3	75.0	
Chemotherapy					
Yes	34	18.6	149	81.4	.001
No	1	1.7	58	98.3	
Radiotherapy					
Yes	14	11.5	108	88.5	.204
No	21	17.5	99	82.5	
Surgical therapy					
Yes	31	14.2	187	85.8	.760
No	4	16.7	20	83.3	
Referral to palliative care					
Yes	21	26.6	58	73.4	<.001
No	14	8.6	149	91.4	
Diabetes mellitus					
Yes	24	15.0	136	85.0	.848
No	11	13.4	71	86.6	
Hypertension					
Yes	24	15.8	128	84.2	.460
No	11	12.2	79	87.8	
Dyslipidemia					
Yes	23	17.7	107	82.3	.144
No	12	10.7	100	89.3	
Chronic cardiac disease					
Yes	7	15.9	37	84.1	.813
No	28	14.1	170	85.9	
Chronic pulmonary disease					
Yes	6	15.8	32	84.2	.803
No	29	14.2	175	15.8	
Chronic kidney disease					
Yes	4	11.1	32	88.9	.619
No	31	15.0	175	85.0	
Liver cirrhosis					
Yes	1	3.0	32	97.0	.059^b^
No	34	16.3	175	83.7	
Arterial thrombosis*					
Yes	4	17.4	19	82.6	.754
No	31	14.2	188	85.8	
Survival status					
Dead	26	25.7	75	74.3	<.001
Alive	9	6.4	132	93.6	
Cause of death					
Infection/sepsis	4	23.5	13	76.5	.580^a^
Cardiopulmonary arrest	14	32.6	29	67.4	
Multiorgan failure	4	30.8	9	69.2	
Disseminated intravascular coagulation/bleeding	0	0.00	2	100.0	
Cancer‐related nonspecific cause	4	16.0	21	84.0	

*Note*: Coronary artery disease, along with congestive heart failure, was included with “chronic cardiac disease.

* Arterial thrombotic events include stroke and peripheral vascular disease but not coronary artery disease.

^a^ *p*‐value was generated by Fisher's Exact test.

^b^ One‐sided *p*‐value was significant at .029.

**TABLE 5:** Comparison of the Mean ± SD of important laboratory values at MPMs diagnosis between patients who had VTE versus patients who had not

Laboratory investigation	VTE	Non‐VTE	*p*‐value
Hemoglobin level (gm/L) at MPMs diagnosis	105 ± 19.3	118 ± 23.6	.003/.001*
Platelet count (×10^9^/L) at MPMs diagnosis	294 ± 235	283.9 ± 150.3	.740/.706*
CRP level (mg/L) at MPMs diagnosis	279.3 ± 121.8	66.3 ± 69.8	.036/.004*
ESR level (mm/h) at MPMs diagnosis	63.8 ± 32.9	53.8 ± 34.3	.199/.143*
LDH level (U/L) at MPMs diagnosis	298.3 ± 221	256.6 ± 138.7	.171/.164*
D‐Dimer level (μ/ml) at MPMs diagnosis	5.0 ± 8.2	4.3 ± 7.0	.723/.476*

Abbreviations: CRP, C‐reactive protein, ESR, erythrocyte sedimentation rate, LDH, lactate dehydrogenase.

* The *p*‐values with asterisk sign were generated by Mann–Whitney test.

### 3.3 Intensive care unit admission and risk of VTE in MPMs

A small fraction (19.0%, *n* = 46) of the cases were admitted to the ICU after MPMs diagnosis for various reasons. Few patients needed intubation with mechanical ventilation (*n* = 27) and vasopressors (*n* = 34). Upon ICU admission, over a half (58.7%, *n* = 27) received prophylactic anticoagulants with enoxaparin (*n* = 14) or unfractionated heparin (*n* = 13). Regardless of the type and dose of prophylactic anticoagulants, ICU admission was significantly associated with higher VTE (*p* = 0.001) (Table 6).

**TABLE 6:** Comparison of ICU admission, in‐ICU complications, and prophylactic anti‐coagulants in patients who developed VTE versus Non‐VTE

	VTE		Non‐VTE		
	*N*	%	*N*	%	*p*‐value
Intensive care unit admission					
Yes	14	30.4	32	69.9	.001
No	21	10.7	175	89.3	
Mechanical ventilation					
Yes	7	25.9	20	74.1	.522
No	7	36.8	12	63.2	
Vasopressors/inotropes use					
Yes	12	35.3	22	64.7	.294
No	2	16.7	10	83.3	
Prophylactic anticoagulant during intensive care unit admission					
Yes	9	33.3	18	66.7	.749
No	5	26.3	14	73.7	
Low molecular weight heparin					
Yes	5	35.7	9	64.3	1.000*
No	4	30.8	9	69.2	
Unfractionated heparin					
Yes	4	30.8	9	69.2	1.000*
No	5	35.7	9	64.3	

* The *p*‐values with the asterisk sign were generated by Fisher's Exact test.

### 3.4 Predictors of VTE after MPMs diagnosis based on binary logistic regression

On univariate logistic regression analysis, the predictors of VTE after MPMs were female gender (OR = 2.47, 95% CI 1.132–5.414, *p* = .023), history of VTE prior to MPMs diagnosis (OR = 5.91, 95% CI 1.85–18.85, *p* = .003), ECOG performance status at MPMs diagnosis (OR = 1.66, 95% CI 1.03–2.65, *p* = .036), non‐metastatic versus metastatic first primary malignancy (OR = 0.328, 95% CI 0.140–0.765, *p* = .010), hemoglobin level at MPMs diagnosis (OR = 0.975, 95% CI 0.96–0.99, *p* = .004), CRP level at MPMs diagnosis (OR = 1.023, 95% CI 1.001–1.05, *p* = .036), chemotherapy (OR = 13.23, 95% CI 1.78–98.94, *p* = .012), ICU admission after MPMs diagnosis (OR = 3.646, 95% CI 1.69–7.90, *p* = .001), and no referral versus referral to palliative care (OR = 0.260, 95% CI 0.124–0.545, *p* < .001). On multivariate logistic regression analysis, the predictors of VTE after MPMs diagnosis were non‐smoking versus smoking (OR = 0.214, 95% CI 0.54–0.856, *p* = .029), non‐metastatic versus metastatic first primary malignancy (OR = 0.121, 95% CI 0.057–0.256, *p* < .001), cirrhosis versus no cirrhosis (OR = 0.118, 95% CI 0.015–0.943, *p* = .044), ICU admission after MPMs diagnosis (OR = 14.442, 95% CI 2.076–100.460, *p* = .007), and ECOG performance status of two compared with 0 at the time of MPMs diagnosis (OR = 26.850, 95% CI 1.676–430.049, *p* = .020) (Table 7).

**TABLE 7:** Multivariate logistic regression model examining the predictors of VTE after MPMs diagnosis

	Odds ratios (95% confidence interval)	*p*‐value
Gender		
Female	0.550 (0.785–0.355)	.166
Male	Ref.	
Smoking		
Non‐smoker	0.214 (0.54–0.856)	.029
Smoker	Ref.	
History of venous thromboembolism		
Yes	3.835 (0.985–14.926)	.053
No	Ref.	
Cirrhosis		
Yes	0.118 (0.015–0.943)	.044
No	Ref.	
Dyslipidemia		
Yes	1.086 (0.498–2.367)	.835
No	Ref.	
Chronic antiplatelet use		
Yes	2.259 (0.820–6.223)	.115
No	Ref.	
Chemotherapy		
Yes	0.857 (0.372–1.976)	.718
No	Ref.	
Metastasis of the first primary malignancy		
No	0.121 (0.057–0.256)	<.001
Yes	Ref.	
Intensive care unit admission		
Yes	9.942 (1.940–50.953)	.006
No	Ref.	
Referral to palliative care		
Yes	1.886 (0.881–4.038)	.102
No	Ref.	
ECOG performance status at MPMs diagnosis^a^		
3	2.958 (0.076–114.537)	.561
2	26.850 (1.676–430.049)	.020
1	3.547 (0.405–31.038)	.253
0	Ref.	Ref.

Abbreviations: ECOG, eastern cooperative oncology group; MPMs, multiple primary malignancies.

^a^ ECOG performance status 4 was not included in the model due to the small number of patients (*n* = 3).

## 4 DISCUSSION

Cancer‐associated thrombosis is a multifactorial highly complicated process that is considered a major cause of morbidity and mortality.^1^, ^2^, ^3^ Compared with arterial thrombosis, which is observed in 1% to 4.7% of cancer patients, VTE is observed in up to 20% of cancer patients and represents a notable financial burden on the health care system.^29^ In fact, it is estimated that up to 30% of all VTEs are cancer‐related,^30^ and up to 20% of VTE‐associated mortality are seen in cancer patients.^4^, ^5^ The risk of VTE in cancer patients can be four to sixfold higher than in patients without cancer,^15^ with a cumulative incidence of 1%–8% depending on the studied patients, cancer type, and associated factors.^30^ The purpose of this study was to gain insight about the burden of VTE in patients with at least two biopsy‐proven malignancies. We particularly aimed to assess the rate of VTE among MPMs and develop predictors for VTEs and their associated mortality. By achieving these objectives, we identified whether the rate of VTE in patients with MPMs is higher than in those with a single malignancy and whether VTE can be predicted by specific clinical and laboratory parameters at MPMs diagnosis.

In the present study, we identified 36 VTE events after the second primary malignancy diagnosis in 35 patients (14.5%). Although there are no studies addressing VTE in patients with MPMs to compare with ours, data from a systemic review and meta‐analysis suggest an annual incidence of 0.5%–20.5%. This wide range signifies the presence of great heterogeneity as some cancers, such as stomach, pancreatic, and brain cancers, are associated with a higher risk of VTE compared to other low‐risk cancers. To further clarify, the annual incidence of first VTE ranges from 10% to 15% in lung, brain, ovarian, pancreatic, and stomach cancers but 3%–5% in other types of cancer.^31^ Because of that, our results are highly heterogeneous, which is a major limitation in our study. In our inclusion criteria, we included patients who had at least 3 months of follow‐up after MPM diagnosis, which is a great limitation in our study. Regardless, 14.5% is at the upper limit but within the range of previously published data. Even after adjusting the inclusion criteria to 1 year instead of 3 months, the percentage of VTE increased to 15.3% which is still within the range mentioned in the aforementioned systemic review and meta‐analysis.^5^

Platelets are highly implicated in cancer‐associated thrombosis.^32^ Tissue factor (TF), a transmembrane protein, primarily initiates the extrinsic limb of the coagulation pathway, activates platelets and, therefore, initiates clotting. This has been associated with VTE and worse outcomes in pancreatic cancer.^33^ Microparticles increase the expression of TF which, in turn, activates platelets and accelerates thrombosis.^34^ Through the C‐type lectin receptor‐2, malignant endothelial cells express a protein, known as Podoplanin, that activates platelets and accelerate thrombosis although the role of Podoplanin in cancer‐associated thrombosis was only described in intracranial malignancies.^33^, ^35^ In addition, some malignant cells secrete mucin, which is known to be thrombogenic as it activates and aggregates platelets via adenosine diphosphate and thrombin.^36^ This is especially true in hypoxic environment, as endothelial cells synthesize platelet‐activating factor, which is a strong platelet agonist and neutrophils activator.^33^ Recently, it is thought that neutrophil extracellular trap, which forms as a result of reactive oxygen species in response to malignant cells engulfment and release of intracellular materials, plays an important role in cancer‐associated thrombosis.^37^ Coagulation cascade and fibrinolysis are also affected in cancer patients. To emphasize, although this was only proven in pancreatic cancer, antithrombin III, heparin cofactor II, protein C and S, and thrombomodulin levels decline as the disease progresses. Moreover, malignant cells highly express PAI‐1, an inhibitor of fibrinolysis, predisposing thrombotic events.^33^

Inflammation is an additional thrombogenic factor in cancer.^38^ Dying malignant cells secrete damage‐associated molecular patterns initiating an immune response via recognition by pattern recognition receptors, which ultimately lead to a chronic inflammatory response.^33^ This is a segue way into the inflammatory cytokines, such as tumor necrosis factor‐alpha (TNF‐α) and interleukin‐1 (IL‐1), and their role in thrombogenesis. TNF‐α and IL‐1 are known to be strong inflammatory cytokines but can also be thrombogenic by inducing PAI‐1 production, TF and von Willebrand factor release and downregulating thrombomodulin.^33^, ^38^

We found that elevated CRP level at the time of MPMs diagnosis could predict VTE (*p* = .036) but not VTE mortality (*p* = .457) though literature identifies elevated CRP as a predictor of early mortality in cancer patients.^39^ CRP is a plasma protein that is synthesized in the liver, induced by IL‐1, IL‐6, and TNF‐α, and considered a marker for inflammation, infection, and tissue injury. It is known that CRP is a marker for atherothrombosis and cardiovascular events.^40^ This supports its role in thrombogenesis in addition to TNF‐α and IL‐1, which induce its synthesis, role. We did not find elevated ESR level as a predictor for VTE (*p* = .199) or its associated mortality (*p* = .785) though some studies found elevated ESR as a marker for worse survival, but not VTE, in cancer patients.^41^, ^42^, ^43^

In addition to the aforementioned, cancer patients need frequent hospitalization, multiple surgical interventions, immobilization, central and peripheral vascular catheters, chemoradiation, and have a higher risk of infection, all of which contribute to the plethora of thrombosis.^21^ In the present study, chemotherapy but not surgery or radio‐therapy was associated with VTE (*p* = .001, .760, and .204, respectively). We did not collect the type of chemotherapy used, which is another limitation in our study, and we did not find an association between VTE and the number of lines (*p* = .282). Several cancer therapies have been linked to VTE. Roughly a quarter of patients treated with Cisplatin‐based chemotherapy, for example, develop thrombotic events within 4 weeks.^18^ Moreover, two cases of cerebral sinus thrombosis after Cisplatin‐based chemotherapy initiation were reported.^44^, ^45^ Other agents that are known to be thrombogenic include Fluorouracil, Lenalidomide, Thalidomide, Asparaginase, epidermal growth factor receptor inhibitors such as Cetuximab and Panitumumab, angiogenesis inhibitors such as Bevacizumab and Sunitinib, cyclin‐dependent kinase inhibitors such as Ribociclib and Abemaciclib, and hormonal therapy such as Tamoxifen and aromatase inhibitors.^18^, ^19^, ^20^, ^21^, ^46^, ^47^

Three‐quarters (75.6%) and a half (50.4%) of the included patients were treated with chemotherapy and radiotherapy, respectively. Chemotherapy with or without radiotherapy often induces anemia via myelosuppression and bone marrow ablation.^48^ Furthermore, even before chemoradiation, the majority of our patients have multiple comorbid conditions, such as DM, HTN, CKD, and IHD, and might be anemic due to anemia of chronic disease. We found low hemoglobin (anemia) as a predictor for VTE (*p* = .004). We believe that the probable reason behind this is the use of erythropoietic agents as a treatment of chemotherapy‐induced anemia although we have not looked into it, which is another limitation in our study. Erythropoietic agents have been associated with both venous and arterial thromboembolic events, and they have been well‐studied, particularly among cancer patients.^49^, ^50^, ^51^, ^52^ To emphasize, in a study that included 4610 patients treated with erythropoietin stimulating agents and 3562 control patients, the relative risk for VTE was 1.57.^50^ Published data have also suggested higher mortality rates among cancer patients treated with erythropoietin stimulating agents.^49^, ^50^, ^51^, ^52^ It is important to notice that these events are not solely limited to oncology patients because erythropoietin‐stimulating agents have been associated with polycythemia, hypertension, thrombocytosis, platelet hyperactivity, and activation of blood coagulation, all of which are thrombogenic.^53^, ^54^ Although patients with proven genetic mutations that predispose thromboses, such as factor V Leiden, antithrombin, and protein C and S deficiencies, were excluded, only a minor number of the whole sample were tested for these mutations, making this a major limitation in our study.

The ECOG scale is a routinely used prognostic tool developed to assess the functional status and help in the choice of treatment algorithms for cancer patients.^55^, ^56^ Patients are given a score of 0–4, where zero means the patient is fully ambulatory, and four means the patient is fully bedridden.^55^ In this study, we found a significant association between VTE and higher ECOG score at the time of MPMs diagnosis (*p* = .003). This is expected as reduced performance status means increased immobility, which indicates stasis of blood flow. We found referral to palliative care as a predictor for VTE (*p* < .001) and VTE‐associated mortality (*p* = .001). This is also largely explained by the fact that patients with reduced performance status and severe disease that is not responsive to treatment would be referred to palliative care. Although more than half of the patients admitted to the ICU received prophylactic anticoagulants in the form of enoxaparin or unfractionated heparin, ICU admission was identified as a predictor of VTE (*p* = .003). Critically ill patients are at increased risk for VTE due to their comorbidities, reason of admission such as multi‐organ failure, sepsis, and trauma, prolonged immobilization which is further potentiated by sedation, central or peripheral venous catheterization, and invasive procedures.^56^, ^57^, ^58^, ^59^

It is known that patients with MPMs, compared to single malignancy,^60^ and synchronous malignancy, compared with metachronous malignancy, have worse survival.^28^, ^61^, ^62^, ^63^ Initially, malignancy weakens the immune system, but subsequently triggers the immune system via the cytokines and the release of growth factors, resulting eventually in an exhausted and weak immune system. There is a possibility that the strengthened immunity is the cause of the longer survival in patients with metachronous MPMs. However, in patients with synchronous MPMs, due to the short interval, the immune system is seriously compromised and cannot be triggered and strengthened, compromising the survival.^61^ We did not find an association between MPMs category (synchronous or metachronous) and VTE (*p* = .153). In our study, the median survival time was 1.9 ± 0.6 and 7.4 ± 1.5 years for patients with VTE and without, respectively, and the difference was highly statistically significant (*p* < .001). Regardless of MPMs as, to the best of our knowledge, no studies concerning VTE in MPMs were done, it is well‐described that VTE compromises cancer patients' survival and complicates their disease courses as it increases the risk of bleeding and commonly reoccurs, and so, in general, our results are in agreement with literature.^64^, ^65^, ^66^

It is well‐known that high doses of glucocorticoids can contribute to the development of arterial thrombotic events. However, it is still debatable whether glucocorticoids increase the risk of VTE.^67^, ^68^ Knowing the fact that patients with Cushing's syndrome, who have prolonged exposure to glucocorticoids, have an increased risk of VTE can further support the relation between glucocorticoids and VTE. Many studies have been done to investigate this relation and found that patients with inflammatory diseases such as asthma, chronic obstructive pulmonary disease, arthritis, and autoimmune diseases have a higher incidence of VTE. Nevertheless, this high incidence could be also caused by the disease itself.^67^, ^68^ We could not find an association between glucocorticoids and VTE in our study, which is probably attributed to the small number (*n* = 26) of patients who were on glucocorticoids.

The current findings indicate that patients with MPMs should be considered a high‐risk group for VTE. The impact of cancer will continue to increase as the number of people surviving cancer increases, as well as the risk for subsequent primary cancers. An important contribution of the current findings is considering pharmacological thromboprophylaxis in the studied population, especially in those with a history of VTE prior to MPMs diagnosis, metastatic first primary malignancy, severe anemia, elevated CRP, poor ECOG at the time of MPMs diagnosis, and patients on chemotherapy. In terms of future research, it would be useful to apply a more restricted inclusion and exclusion criteria, include erythropoietin stimulating agents, chemotherapy agents, and oral contraceptive use, and increase the sample size by conducting a multicenter study to improve and further solidify the findings of this study. Despite these limitations, the present study enhanced our understanding of the risk of VTE among patients with MPMs. We hope that the current research will encourage future researchers to investigate this important area.

## 5 CONCLUSION

To the best of our knowledge, the present study represents the first attempt to address VTE in MPMs and provides predictors of thrombosis and thrombosis‐associated mortality in MPMs. VTE was diagnosed in 14.5% of patients with MPMs and it significantly compromises their survival. Predictors of VTE after MPMs were female gender, history of VTE prior to MPMs diagnosis, metastatic first primary malignancy, poor performance status, anemia, elevated CRP level at MPMs diagnosis, ICU admission after MPMs diagnosis, chemotherapy, and referral to palliative care. We believe that these results might be of particular benefit since the phenomenon of multiple primary malignancies is becoming more frequently encountered.

## AUTHOR CONTRIBUTIONS

**Moustafa S. Alhamadh:** Conceptualization (lead); data curation (equal); funding acquisition (equal); methodology (equal); project administration (equal); supervision (lead); writing – original draft (lead); writing – review and editing (lead). **Rakan B. Alanazi:** Data curation (equal); writing – original draft (supporting); writing – review and editing (equal). **Muhannad Q. Alqirnas:** Data curation (supporting); writing – original draft (supporting); writing – review and editing (equal). **Abdulrahman Yousef Alhabeeb:** Methodology (supporting); writing – original draft (equal); writing – review and editing (equal). **Yusra Sajid Chachar:** Data curation (lead); formal analysis (lead); methodology (supporting); writing – review and editing (equal). **Mohammad Omar Farouq Alkaiyat:** Funding acquisition (lead); project administration (lead); resources (equal). **Fouad Sabatin:** Conceptualization (supporting); funding acquisition (supporting); project administration (supporting); supervision (lead); writing – original draft (supporting); writing – review and editing (lead).

## CONFLICT OF INTEREST

The authors have stated explicitly that there are no conflicts of interest in connection with this article.

## ETHICS STATEMENT

The study was approved by the Institutional Review Board of King Abdullah International Medical Research Center, Ministry of National Guard‐Health Affairs, Riyadh, Kingdom of Saudi Arabia (NRC22R/083/02). Informed consent was waived due to the retrospective nature of the study. Access to the data was restricted to the researchers. The confidentiality of all patients was protected, and no names or medical record numbers were used. Privacy and confidentiality were assured and all the data, both hard and soft copies, were kept in a secure place within the National Guard‐Health Affairs premises.

## Data Availability

The data used to write this article are available upon request from the corresponding author.
